# National Association of Dental Faculties

**Published:** 1904-10

**Authors:** J. E. Storey

**Affiliations:** Morenci, Ariz.


### Domestic Correspondence.

### NATIONAL ASSOCIATION OF DENTAL FACULTIES.

I do not know that I have any right to give expression in opposition to the amount of fuss that has emanated from many of the colleges of late on account of the change of time from three to four years. It certainly looks strange to an outsider, one who is without partisan feeling for any of the colleges, and one who can see without the use of a field-glass just what the end will be if harmony is not restored. The colleges who have, and those who intend to, cut loose from the Association of Dental Faculties should be placed on a black list, and at no time during their existence should they be allowed to return to the fold. Their existence will be more than short. Students cannot and will not enter a college whose diploma is not recognized by the Association of Dental Faculties. Our dental laws in the majority of the States demand diplomas from colleges that are members of the Association. The falling off of students from these colleges means the closing of doors. To those colleges that have remained loyal will come the students who seek the higher dental education and a diploma which gives a standing and carries a professional dignity on the face of it. While we will be compelled to admit that the falling off in the attendance will be large and the loss to some of the colleges considerable, yet what the colleges lose the profession gains. For it will gain men who come well grounded in the English language, men who will have the interests of the profession at heart, and men who will work to place the standard as high as it is possible to reach. It means the closing of the dental parlors, protecting the laity and the profession.

The colleges should welcome the day when the weak and puny ones elect to go. The world demands the survival of the fittest. We need strong colleges and honest men to handle them. Men who can be turned by a key of gold are not fit to stand in a lecture-hall. If men of that stamp are allowed to fill the chairs, they will surely contaminate the student and open the gates of empiricism. We would not permit a minister of the gospel to preach other than holy writ from the pulpit. Then why allow our dental colleges to permit men to stand before a body of students and inculcate into them principals other than pertains directly to dentistry, not so much by word of mouth as by actions, and actions often speak volumes. To me dentistry is a hol) calling. Do we not enter the homes of our patients as no other can except the family physician? Were we libertines and charlatans, would we be given the respect due our calling? As we conduct ourselves so are we judged. Then let us stand for higher education, moral principles, and ethical conduct at all times and in all places.

J. E. STOREY.

MORENCI, ARIZ.

